# Zonulin levels in children with chronic immune thrombocytopenia

**DOI:** 10.1097/MD.0000000000045161

**Published:** 2025-10-17

**Authors:** Meriban Karadoğan, Fatma Turkan Mutlu, Veysel Gok, Derya Koçer, Şuayip Keskin

**Affiliations:** a Department of Pediatric Hematology and Oncology, Kocaeli University, Kocaeli, Türkiye; b Department of Pediatric Hematology and Oncology, Kayseri City Hospital, Kayseri, Türkiye; c Department of Pediatric Hematology and Oncology, Erciyes University, Kayseri, Türkiye; d Department of Clinical Biochemistry, Kayseri City Hospital, Kayseri, Türkiye.

**Keywords:** biomarker, children, chronic immune thrombocytopenia, zonulin levels

## Abstract

Chronic immune thrombocytopenia (cITP) is an autoimmune disease characterized by a persistently low circulating platelet count below 100 × 10^9^/L for more than 1 year. Increased intestinal permeability has been implicated in autoimmune diseases due to microbial translocation and disrupted tight junctions. This study aimed to investigate the potential association between zonulin levels, a biomarker of intestinal permeability, and cITP. This case-controlled study included 41 patients with cITP and 41 healthy subjects recruited from a single center in Turkey. All participants were tested for stool *Helicobacter pylori* antigen (Hpa), antinuclear antibody (ANA), anti-dsDNA antibody, and immune profiling. Serum zonulin levels were measured using a human zonulin enzyme-linked immunosorbent assay (ELISA) kit (Elabscience, Houston) with a measurement range of 0.78 to 50 ng/mL. The mean age of cITP patients and control group was 9.6 ± 4.4 and 10.2 ± 4.4 years, with 29 (70.7%) and 22 (53.7%) being female, respectively. Platelet counts in the cITP group were highly variable, ranging from 4 to 127 × 10^9^/L. ANA positivity was detected in 8 patients (19.5%), stool Hpa positivity in 3 patients (7.3%), and systemic lupus erythematosus in 1 patient (2.4%). Serum zonulin levels were significantly higher in cITP patients compared to controls (19.4 vs 9.1 ng/mL; *P* < .001). Patients with cITP exhibited significantly elevated zonulin levels, suggesting a potential link between increased intestinal mucosal permeability and cITP.

## 1. Introduction

Chronic immune thrombocytopenia (cITP) is an autoimmune disorder characterized by a low circulating platelet count below 100 × 10^9^/L that persists for more than a year after onset in about 20% to 25% of children who experience acute ITP caused by destruction of antibody-sensitive platelets in the reticuloendothelial system.^[1,2]^ In addition, cITP in adolescents tends to have a more severe and persistent course compared to younger children, who often experience spontaneous remission.^[1,2]^ Increased intestinal permeability (IP), genetic composition, and environmental factors, such as antigens, macromolecules, nutrients like gluten, toxins, and bacteria, have all been proposed as key players in the pathophysiology of chronic inflammatory diseases, which include metabolic, autoimmune, and allergic disorders.^[3,4]^ Gut epithelial cells are connected by tight junctions (TJ) that maintain the integrity of the first line of defense, the gut barrier against infectious, toxic and allergenic pathogens and exogenous agents. Changes in the gut barrier and IP, termed leaky gut, may allow environmental entities to translocate from the intestinal lumen into the bloodstream and induce the initiation and development of autoimmune diseases.^[3–5]^

Zonulin is a physiological mediator. It is a protein of 47 Kd that regulates IP through modulation of intracellular TJ and has been implicated as a biomarker of IP in autoimmune, metabolic, and neuroinflammatory diseases and cancer.^[6,7]^

In the present study, based on the putative relationship between IP and autoimmunity, we aimed to investigate the relationship between circulating zonulin levels and childhood cITP.

## 2. Materials and methods

This was a case-controlled study that included pediatric patients with cITP and an epual number of healthy controls. The patients attended a single referral center in Turkey. All patients were investigated for stool Helicobacter pylori antigen (Hpa), antinuclear antibody (ANA), anti-double-stranded DNA (dsDNA) antibody, and underwent immune profiling and viral serology testing during diagnosis. Endomysium antibody and tissue transglutaminase were used to rule out celiac disease, which is linked to increased IP and may confound results. The zonulin levels in the patients and controls were compared.

Patients who had used corticosteroids in the 3 months prior the study were excluded. Samples were collected during the period when patients had no evidence of infection and no antibiotics were being used. Venous blood samples were collected in test tubes without anticoagulant for determination of zonulin. The blood samples were centrifuged at 4000 rpm for 10 minutes; then, the sera were separated and stored at −80 ◦C until analysis.

Serum zonulin levels were measured by human zonulin enzyme-linked immunosorbent assay (ELISA) kit with a measurement range of 0.78 to 50 ng/mL (Elabscience, Houston). The zonulin testing was performed according to the manufacturer’s instructions and expressed as ng/mL. Sample concentrations were calculated from calibration curves obtained from study standards of known concentrations. The intra- and interassay coefficients of variation were >10%.

This study follows the STROBE guidelines for observational case-control studies. The present retrospective study was approved by the Ethics Committee of Kayseri City Hospital (Date: May 18, 2022, Number: 640) and was conducted in accordance with the ethical standards of the Declaration of Helsinki. Written informed consent was also obtained from the parent(s) of each subject prior to inclusion in the study.

### 2.1. Statistical methods

Mean, standard deviation, median, minimum and maximum values, and frequency and ratio values were used, as appropriate, in the descriptive statistics of the data. The distribution of the variables was measured using the Kolmogorov–Smirnov test. The Mann–Whitney *U* test was used in the analysis of quantitative independent data. The chi-square test was used when analyzing qualitatively independent data. The effect size was examined using the receiver operator characteristics curve analysis. SPSS version 28.0 was used for analysis (IBM Inc., Armonk).

## 3. Results

A total of 41 cases of cITP with a mean age of 9.6 ± 4.4 years, of whom 29 (70.7%) were females, were recruited. The control group consisted of 41 healthy children with a mean age of 10.2 ± 4.4 years, of whom 22 (53.7%) were female. The groups were similar for age and gender. The demographics, and clinical features of patients are summarized in Table 1.

**Table 1:** The demographics and clinical features of patients.

		Min–max	Median	Mean±SD/n-%
Age/year		2.5–17.6	9.0	9.9 ± 4.4
Gender	F	–	–	29/70.7%
	M	–	–	12/29.3%
PLT/x10^9^/L	–	4.0–127.0	56.0	59.0 ± 27.6
MPV	–	9.2–13.8	11.0	11.2 ± 0.9
ANA(+)	–	–	–	8/19.5%
HP(+)	–	–	–	3/7.3%
dsDNA(+)	–	–	–	1/2.4%
Eltrombopag	(−)	–	–	33/80.5%
	(+)	–	–	8/19.5%

ANA = antinuclear antibody, dsDNA = double-stranded DNA, HP = helicobacter pylori, MPV = mean platelet volume, PLT = platelet.

In the patient group the platelet count ranged from 4 × 10^9^/L to 127 × 10^9^/L with a mean of 59 ± 27.6 × 10^9^/L. ANA positivity was identified in 8 (19.5%), stool Hpa positivity in 3 (7.3%), and ds DNA positivity in 1 (2.4%) patient. Furthermore, 8 (9.5%) patients were using eltrombopag.

**Figure 1.**
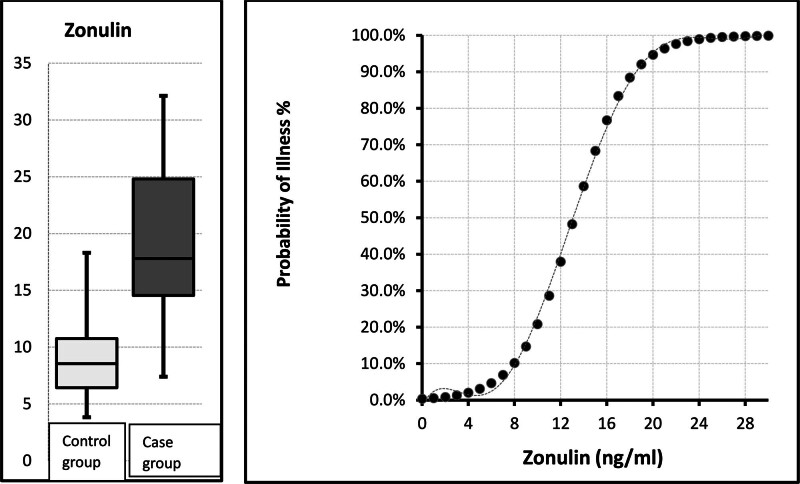
A significant area under the curve was observed for zonulin in separating the patient and control groups. Sensitivity at a zonulin cutoff value of 12 ng/ml was 99.8%, positive predictive value was 99.9%, specificity was 89.7%, and negative predictive value was 85.4%.

The mean zonulin value in the case group (19.4 ± 6.4 ng/mL) was significantly higher than in the control group (9.1 ± 3.6 ng/mL) and the ranges were significantly different (3.8–32.1 vs 3.2–18.1) (*P* <.05, see Table 2 and Fig. 1). Receiver operator characteristics analyze showed that a significant area under the curve of 0.926 (0.869–0.982) for zonulin when separating the patient and control groups. At the zonulin cutoff value of 12 ng/Ml, the sensitivity was 99.8%, the positive predictive value was 99.9%, the specificity was 89.7%, and the negative predictive value was 85.4% (Table 3). Of note, zonulin levels were significantly higher in the over 13 years-old age group than in the under 13 year-old age group. Howewer, zonulin levels did not differ between boys and girls (Table 4).

**Table 2:** Zonulin levels in cases and controls.

		Control group		Case group		*p*
		Mean ± SD/n-%	Median	Mean ± SD/n-%	Median	
Age		10.2 ± 4.4	10.0	9.6 ± 4.4	8.0	0.476*
Gender	F	22/53.7%	–	29/70.7%	–	0.111^X²^
	M	19/46.3%	–	12/29.3%	–	
Zonulin (ng/mL)		9.1 ± 3.6	8.5	19.4 ± 6.4	17.8	**<0.001** *

mL = milliliter, ng = nanogram, SD = standard deviation.

* Mann–Whitney *U* test/^X²^ Chi-square test.

**Table 3:** ROC analysis for zonulin.

		Control group	Case group	Sensitivity	Positive Prediction	Specificity	Negative Prediction
Zonulin (ng/ml)	≤12	35	6	99.8%	99.9%	89.7%	85.4%
	>12	4	37				

mL = milliliter, ng = nanogram, ROC = receiver operating characteristic.

**Table 4:** Zonulin levels by gender and age group.

		Zonulin (ng/mL)			*P*
		Min–max	Median	Mean.±ss	
Age (year)	<13	7.40–32.13	16.81	17.74 ± 6.15	**.008** *
	≥13	12.10–31.16	24.16	23.26 ± 5.62	
Gender	Female	7.40–32.13	17.81	19.39 ± 6.34	.841*
	Male	9.62–31.51	17.69	19.28 ± 6.99	

* Mann–Whitney *U* test.

## 4. Discussion

Weakening of the intestinal barrier which is made up of epithelial cells connected by TJ and regulated by transmembrane proteins such as claudins and occludins and supported junctional integrity and paracellular sealing by actin cytoskeleton and microtubules, may lead to increased exposure of the submucosal immune system cells to pathogens and allergens and may result in autoimmunity.^[3,4]^ Infectious, environmental and hormonal agents and their interactions with host tissue may cause inflammation, and the host immune response has been shown to induce autoimmunity in a variety of ways.^[8,9]^ Changes in IP have been described and zonulin has been studied as a marker in a large number of CIDs including in type 1 diabetes mellitus, obesity-associated insulin resistance, celiac disease, asthma, multiple sclerosis, ankylosing spondylitis, autism spectrum disorders, inflammatory bowel disease, obesity and non-celiac gluten sensitivity.^[10–21]^ The present study found that zonulin levels were significantly higher in patients with cITP than in healthy controls. This finding suggests there may be an effect of a leaky gut in this autoimmune disease. Stool Hpa positivity was identified in only 3 (7.3%) patients with cITP. Inadequate diagnostic testing may explain the low positivity rate in the present study. *H. pylori* may have extra-gastric manifestations, such as hematologic diseases, including cITP, and eradication therapy is recommended in guidelines.^[22,23]^

The molecular mechanisms of *H. pylori*-associated ITP is still unclear and probably involve multiple factors. *H. pylori* components can mimic the molecular structure of platelet antigens and can modulate the Fc-receptor balance of monocytes/macrophages in favor of Fc-receptor activation.^[23,24]^ It has been shown that *H. pylori* infection and small intestinal bacterial overgrowth may coexist. It has been suggested that mucosal atrophy and gastric pH changes predispose patients to small intestinal bacterial overgrowth.^[25]^ In some of our patients with cITP, we have observed high platelet counts after using antibiotics or taking probiotics for other reasons.^[26]^ This finding may also indicate an intestinal dysbiosis. A variety of gut barrier/microbiota disruptors, including diet, infections and stress, may potentially lead to microbial translocation and subsequent local and systemic inflammation. Reversal of intestinal leakage may be an attractive therapeutic strategy for bacterial translocation and potentially CIDs.^[27]^ Hippocrates said, “All disease begins in the gut.’’ Further research is needed to identify these entities and possible associated non-gastrointestinal conditions.^[5]^ Similar work was recently reported in adults^[28]^ that relates to the development and/or persistence of autoimmune thrombocytopenia, but this is the first such study in a pediatric population.

This study has many limitations including no data on gastrointestinal symptoms, no endoscopic analysis, a lack of gut microbiota characterization, no endotoxin assessment, and no measurement of other inflammatory markers such as IL-6, TNF or C-reactive protein. These deficiencies should be adjusted for in future studies of this topic. It is also important to emphasize how the latency of cITP affects IP, positive family history, platelet size, and the reaction to first-line treatments. Additionally, serum creatinine levels were not measured, which could be relevant for correlating protein levels such as zonulin in conditions like hemolytic uremic syndrome.

## 5. Conclusion

To the best of our knowledge, this is the first study examining zonulin levels in pediatric patients with cITP. The results showed that zonulin was significantly higher in children with cITP compared to healthy children. We propose that this reflects an increase in IP that relates to the development and/or persistence of autoimmune thrombocytopenia. Correcting the intestinal barrier defect may have a positive prognostic effect and may be a possible additional alternative approach for controlling cITP. Follow-up studies will be helpful in elucidating the cause-effect relationships, underlying mechanisms, and connections between gut, immune system, and subclinical systemic and local inflammatory bowel disease.

## Author contributions

**Conceptualization:** Meriban Karadoğan, Fatma Turkan Mutlu, Veysel Gok.

**Data curation:** Meriban Karadoğan, Derya Koçer, Şuayip Keskin.

**Formal analysis:** Meriban Karadoğan, Veysel Gok, Derya Koçer.

**Investigation:** Meriban Karadoğan, Fatma Turkan Mutlu, Şuayip Keskin.

**Methodology:** Meriban Karadoğan, Veysel Gok, Derya Koçer.

**Supervision:** Meriban Karadoğan, Fatma Turkan Mutlu, Veysel Gok.

**Validation:** Meriban Karadoğan, Veysel Gok, Şuayip Keskin.

**Writing – original draft:** Meriban Karadoğan, Fatma Turkan Mutlu, Veysel Gok.

**Writing – review & editing:** Meriban Karadoğan, Fatma Turkan Mutlu, Veysel Gok.
